# Understanding GEMIN5 Interactions: From Structural and Functional Insights to Selective Translation

**DOI:** 10.1002/wrna.70008

**Published:** 2025-04-02

**Authors:** Encarnacion Martinez‐Salas, Salvador Abellan, Rosario Francisco‐Velilla

**Affiliations:** ^1^ Centro de Biologia Molecular Severo Ochoa CSIC‐UAM Madrid Spain

## Abstract

GEMIN5 is a predominantly cytoplasmic protein, initially identified as a member of the survival of motor neurons (SMN) complex. In addition, this abundant protein modulates diverse aspects of RNA‐dependent processes, executing its functions through the formation of multi‐component complexes. The modular organization of structural domains present in GEMIN5 enables this protein to perform various functions through its interaction with distinct partners. The protein is responsible for the recognition of small nuclear (sn)RNAs through its N‐terminal region, and therefore for snRNP assembly. Beyond its role in spliceosome assembly, GEMIN5 regulates translation through the interaction with either RNAs or proteins. In the central region, a robust dimerization domain acts as a hub for protein–protein interaction, while a non‐canonical RNA‐binding site is located towards the C‐terminus. Interestingly, GEMIN5 regulates the partitioning of mRNAs into polysomes, likely due to its RNA‐binding capacity and its ability to bind native ribosomes. Understanding the functional and structural organization of the protein has brought an increasing interest in the last years with important implications in human disease. Patients carrying *GEMIN5* biallelic variants suffer from neurodevelopmental delay, hypotonia, and cerebellar ataxia. This review discusses recent relevant works aimed at understanding the molecular mechanisms of GEMIN5 activity in gene expression, and also the challenges to discover new functions.

## Graphical abstract

GEMIN5 structural domains. Overview of GEMIN5's role in snRNP assembly and translation regulatory events.

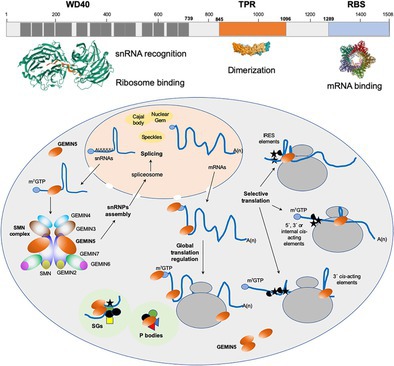

## 1 Introduction: GEMIN5 Overview

GEMIN5 is an abundant cytoplasmic RNA‐binding protein (RBP) known to be involved in the assembly of small nuclear ribonucleoproteins (snRNPs) and translation regulation (reviewed by Martinez‐Salas et al. 2020; Nelson and Pandey 2024). Initially, GEMIN5 was identified as a member of the survival of motor neurons (SMN) complex in human cells (Gubitz et al. 2002). This macromolecular complex is responsible for the assembly of the Sm ring proteins D1/D2/F/E/G into the U‐rich small nuclear (sn)RNAs (Urlaub et al. 2015), generating the snRNP particles (Pellizzoni et al. 2002). These macromolecular entities, also known as U snRNPs, are the critical components of the spliceosome that, ultimately, mediate pre‐mRNA splicing (reviewed in Fischer et al. 2011). Subsequent studies identified GEMIN5 as a factor involved in the regulation of translation of specific mRNAs, and later, as a ribosome‐associated protein (Pacheco et al. 2009; Francisco‐Velilla et al. 2016) (see below).

GEMIN5 is conserved in eukaryotic organisms all along the evolutionary scale (Liao et al. 2025), with 203 known orthologs (http://research.gzsys.org.cn/rbpworld/#/home). In humans, GEMIN5 is encoded by a single gene located on chromosome 5. The primary transcript, about 50 kb long, comprises 28 exons encoding a protein of 1508 amino acids. The protein is ubiquitously expressed (Kim et al. 2014; Uhlen et al. 2015), being clearly detected in the cerebral cortex, cerebellum, colon, kidney, testis, placenta, breast, lymph node, and bone marrow. In metazoan organisms, *GEMIN5* is an essential gene, as shown by the embryonic lethal phenotype of the *Rigor mortis* gene (Gates et al. 2004), the *Drosophila melanogaster* ortholog. Likewise, attempts to generate KO mouse models displayed an embryonic lethal phenotype (Rajan et al. 2022). In addition to the lack of viability of *Gemin5* KO in these animal model systems, it has been recently shown that *GEMIN5* biallelic variants lead to a spectrum of neurodevelopmental diseases that occur to different degrees (Kour et al. 2021; Francisco‐Velilla, Embarc‐Buh, del Caño‐Ochoa et al. 2022; Saida et al. 2021; Zhang et al. 2023; Ibrahim et al. 2023; Cascajo‐Almenara et al. 2024; Zhang et al. 2024), often resulting in neurodevelopmental delay, hypotonia, and cerebellar ataxia (reviewed in Nelson and Pandey 2024).

## 2 Structural Domains

**FIGURE 1:**
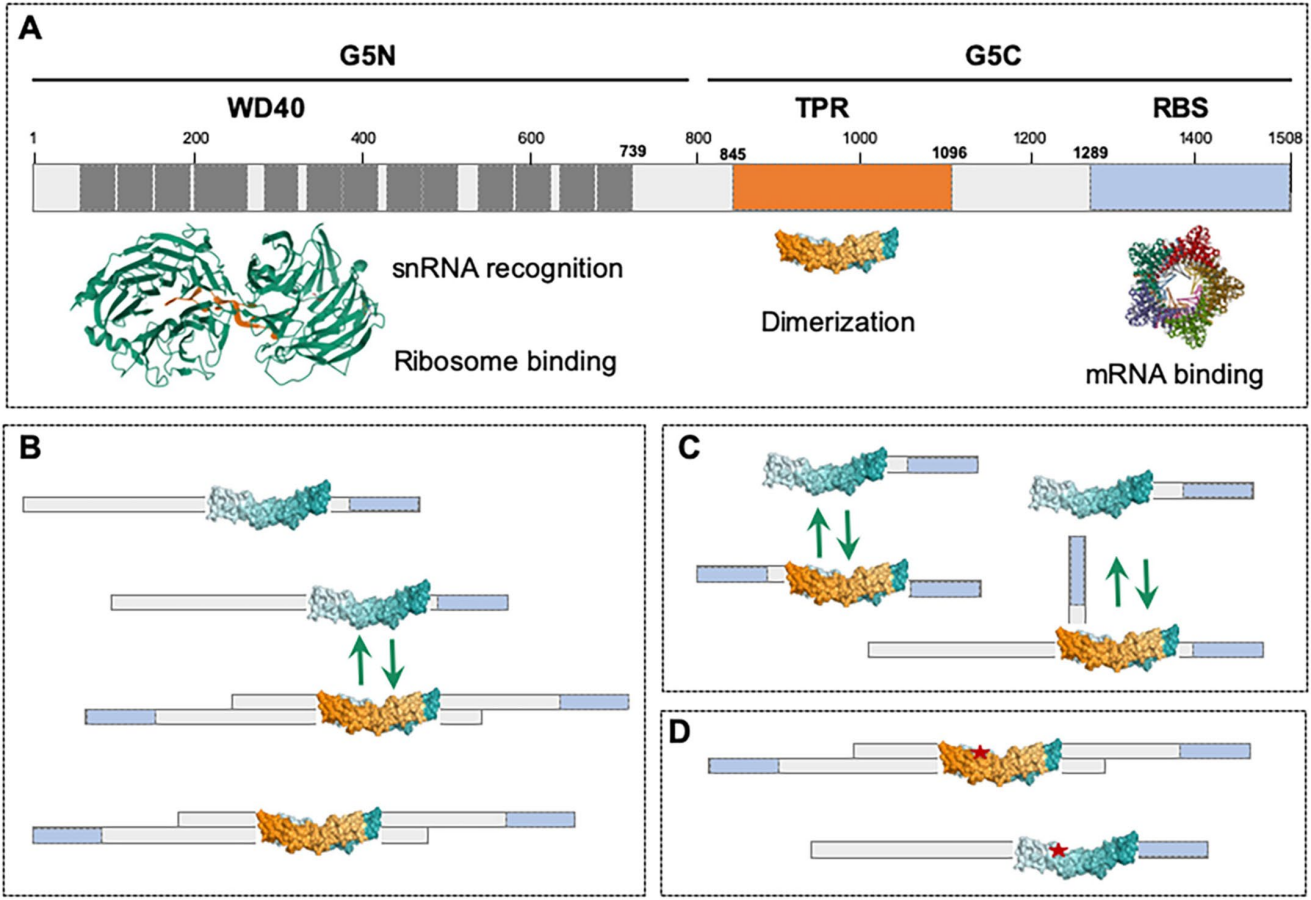
Modular organization of GEMIN5 protein. (A) Structural domains along with their function. The approximate location of the tryptophan‐aspartic acid (WD)40 repeats (dark gray), the tetratricopeptide repeats (TPR) dimerization module (orange), and the RNA‐binding site (RBS) (light blue) are indicated (Gubitz et al. 2002; Xu et al. 2016; Moreno‐Morcillo et al. 2020; Fernandez‐Chamorro et al. 2014; Guo et al. 2022). G5N and G5C stand for N‐terminal and C‐terminal region of GEMIN5, respectively. Numbers indicate the amino acid position; the flanking residues of each domain are shown in bold. The structural models of the WD40 repeats, the TPR dimerization domain, and the pentamer assembled by the RBS domain were downloaded from PDB. (**B**) Schematic cartoon of the monomer and homodimer forms of the full‐length protein. The anti‐parallel orientation of each unit on the homodimer (Moreno‐Morcillo et al. 2020) is further highlighted by the relative position of the orange and blue boxes of the TPR and the RBS domains at the C‐terminus of the protein. In the cellular context, the monomer and the homodimer conformation of the protein, presumably coexisting in equilibrium, is determined by the dimerization ability of the TPR domain. (C) Recruitment of the full‐length GEMIN5 protein by the half C‐terminal region. Schematic view of the monomer and homodimer forms of G5C, the G5C‐GEMIN5 heterodimer, and the monomer and homodimer forms of the full‐length protein, presumably coexisting in the cellular scenario. In this context, the formation of the G5C‐GEMIN5 heterodimer highjacks a fraction of GEMIN5, consequently decreasing the effective concentration of the full‐length protein. (D) Conformational changes in the TPR domain of GEMIN5 mutants. *GEMIN5* variants encoding amino acid substitutions (depicted by a red asterisk) generate defective structural conformations, often combined with protein instability (Kour et al. 2021; Francisco‐Velilla, Embarc‐Buh, del Caño‐Ochoa et al. 2022), likely resulting in unbalanced formation of GEMIN5 homodimers compared to control protein.

The human GEMIN5 protein is organized into distinct functional and structural domains (Figure 1A). The N‐terminal half (G5N) consists of two domains that comprise seven‐bladed WD40 repeats (named WD1, WD2) (PDB: 5TEE, 5TEF, 5H3S, 5H1J, 5H1L), which recognize the Sm site (5′AUUUUU) of snRNAs and the m^7^GTP (cap) structure via base‐specific interactions (Xu et al. 2016; Jin et al. 2016; Tang et al. 2016). The C‐terminal half (G5C) encompasses a dimerization module in the central region and a non‐canonical RNA‐binding site (RBS) at the most C‐terminal end (Fernandez‐Chamorro et al. 2014; Moreno‐Morcillo et al. 2020). The three‐dimensional structure of the dimerization module folds as a tetratricopeptide repeat (TPR) moiety (PDB: 6RNQ, 6RNS), comprising 17 α‐helices. The homodimer is formed by two molecules of the protein, which are located in an antiparallel orientation, resembling the shape of a canoe. CryoEM structural studies of the G5C region unveiled a decamer architecture arranged as a dimer of pentamers (PDB: 7XDT, 7XGR) (Guo et al. 2022), which determines the RNA‐binding and translation regulatory properties of this region.

The TPR module dictates the dimerization capability of the protein (Moreno‐Morcillo et al. 2020; Francisco‐Velilla, Abellan, Embarc‐Buh et al. 2024). Recent studies of the relevance of the TPR domain for GEMIN5 multimerization in mammalian cells revealed that only the regions of the protein harboring a wild‐type TPR moiety are able to recruit the endogenous GEMIN5 protein (Figure 1B,C), strongly suggesting that the protein can adopt the conformation of an oligomer in the cell cytoplasm. These data support the view that the TPR moiety of GEMIN5 plays a key role in promoting the assembly of an oligomer particle in vitro and in living cells of still unknown complexity.

Although the three‐dimensional structures of the separated G5N and G5C regions have been solved (Xu et al. 2016; Guo et al. 2022), the structure of the full‐length protein and the stoichiometry of the monomer units in the oligomer entity assembled in vivo remain unknown. Notwithstanding, the recruitment of the endogenous GEMIN5 protein by the G5C region conveys the idea that the G5C fragment lacking the WD40 repeats domain but maintaining the oligomerization capacity could hijack, at least partially, the full‐length GEMIN5 protein (Francisco‐Velilla, Abellan, Embarc‐Buh et al. 2024) (Figure 1C), presumably interfering with its different activities. Remarkably, loss of protein oligomerization does not perturb the primary cytoplasmic localization of GEMIN5, reinforcing the preferential subcellular location of this protein and, consequently, interfering with its cytoplasmic activities.

It is important to note that a large fraction of the currently known *GEMIN5* pathogenic variants eventually result in amino acid replacements within the TPR module (Kour et al. 2021; Rajan et al. 2022; Francisco‐Velilla, Embarc‐Buh, del Caño‐Ochoa et al. 2022). These mutant versions of the protein have been shown to alter the structural conformation of the TPR moiety, interfering with endogenous GEMIN5 recruitment, presumably due to a lack of GEMIN5 homodimerization (Figure 1D). Moreover, several of these biallelic variants are known to induce conformational changes in the protein structure, likely destabilizing the dimerization capacity of the mutant proteins (Francisco‐Velilla, Embarc‐Buh, del Caño‐Ochoa et al. 2022). In further support of the relevance of the TPR‐like structure for protein function, the recessive mutant on the dimerization module of one allele is often accompanied by either a truncated version, a frameshift, or an intron variant in the other allele, as well as a destabilizing substitution in the WD40 repeats, the TPR module, or the RBS domain. These combinations of biallelic variants presumably result in different partially defective proteins that affect distinct GEMIN5 functions, but still allow partial compensatory activities that eventually lead to disease. Indeed, nowadays there is a spectrum of different neurological disorders associated with *GEMIN5* gene variants (Kour et al. 2021; Rajan et al. 2022; Francisco‐Velilla, Embarc‐Buh, del Caño‐Ochoa et al. 2022; Saida et al. 2021; Zhang et al. 2023; Ibrahim et al. 2023; Cascajo‐Almenara et al. 2024; Zhang et al. 2024). Thus, a better knowledge of the protein activities regarding new functions of each domain, as well as understanding the role of specific residues within them or altered protein levels, is expected to help in the diagnosis of the still poorly characterized illness associated with *GEMIN5* variants.

Despite the work done in the last decade, many questions remain to be addressed concerning the activities of GEMIN5, either individually or in complex with other proteins. In fact, GEMIN5 is often found outside of the SMN complex, alone or in complexes with GEMIN3 and GEMIN4 (Battle et al. 2007), lending support to additional functions of this protein either individually or in association with other factors. Although the AlphaFold model predicts the structure of GEMIN5 as a monomer, currently it is not known which oligomerization state mediates snRNA recognition, mRNA binding, and/or regulation of translation, which are the most documented activities performed by this protein. In addition, although there are previous data showing that the protein is heavily phosphorylated (Husedzinovic et al. 2014; Francisco‐Velilla, Embarc‐Buh, Abellan et al. 2022; https://www.phosphosite.org), in‐depth information on the role of post‐translationally modified versions of the protein is still lacking. All these questions represent the main challenges that need to be addressed in future studies.

## 3 Interactome

RBPs are ubiquitous factors assisting fundamental cellular processes, from genome transcription to splicing, RNA stability, and translation (Hentze et al. 2018). Accordingly, the results obtained from global genomic and proteomic studies (Gerstberger et al. 2014; Caudron‐Herger et al. 2021) reinforce the view that RBPs play key roles in sustaining cell proliferation in all organisms. The organization of GEMIN5 is reminiscent of modular RBPs, which harbor specific domains that confer regulatory activity through interactions with other factors (Van Nostrand, Freese et al. 2020; He et al. 2023). A comprehensive overview of the protein network associated with GEMIN5 underscores a large number of factors related to spliceosome assembly and regulation of translation (Francisco‐Velilla, Abellan, Embarc‐Buh et al. 2024). As shown by exhaustive RNase treatment of the GEMIN5 co‐immunoprecipitated complexes, many of these interactions are driven by RNA bridges, with the marked exception of several ribosomal proteins, which, on the other hand, are consistent with the ribosome binding capacity of GEMIN5 (Francisco‐Velilla et al. 2016).

**FIGURE 2:**
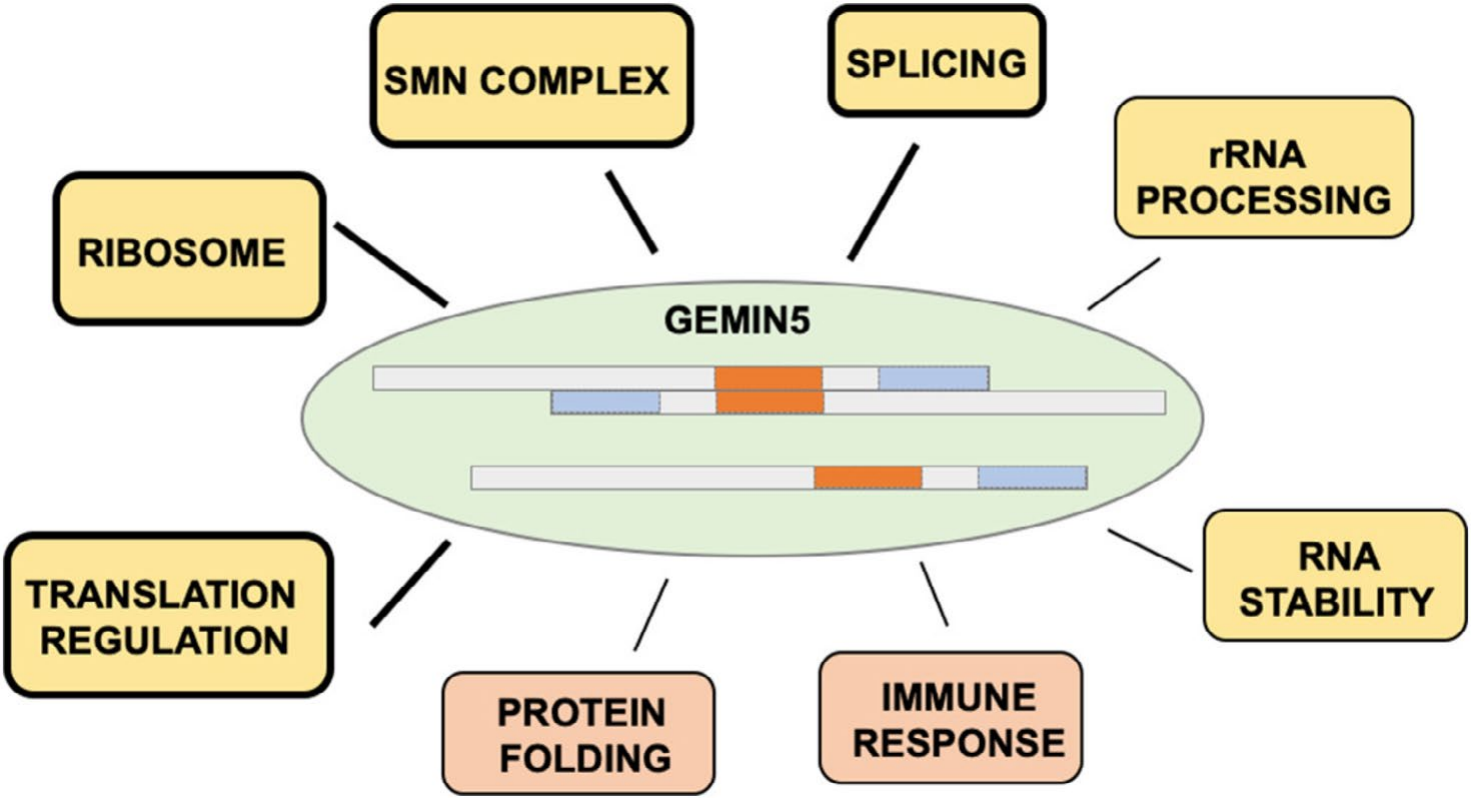
Diagram of the oligomerization‐dependent GEMIN5 association with components of the cellular machinery. The functional gene ontology terms (Translation regulation, Ribosome, SMN complex, Splicing, RNA stability, and rRNA processing) of the oligomerization proficient forms of GEMIN5 (Francisco‐Velilla, Abellan, Embarc‐Buh et al. 2024) are highlighted by yellow rectangles. Thin black lines depict terms identified with lower statistical values (rRNA processing, RNA stability, and Protein folding). Orange rectangles denote biological terms associated with oligomerization deficient GEMIN5 domains (Protein folding, and Immune response).

The oligomerization state of proteins impinges on their direct and indirect targets, which in turn affects their activities in a broad range of cellular processes (Lou et al. 2022; Pérez‐Berlanga et al. 2023). A detailed gene ontology analysis of the factors interacting with different regions of GEMIN5 bearing distinct functional domains reveals a clear relationship between the oligomerization capacity of GEMIN5 and its preferential association with the terms related to the RNA life cycle (Figure 2) (Francisco‐Velilla, Abellan, Embarc‐Buh et al. 2024). The ribosomal proteins identified associated with domains of GEMIN5 promoting dimerization are shown in Table 1. Likewise, factors associated with the terms Splicing, SMN complex, RNA stability, Translation regulation, and RNA processing (Table 2) also depend on the dimerization module of GEMIN5. This is exemplified by proteins performing dedicated functions in RNA stability and translation regulation, such as G3BP Stress Granule Assembly Factor 1 (G3BP1), La Ribonucleoprotein 1 (LARP1), or YTH N6‐Methyladenosine RNA Binding Protein F1 (YTHDF1).

**TABLE 1:** Ribosomal proteins associated with GEMIN5 oligomerization domains.

Ribosomal protein	GEMIN5 interacting domains^a^
RPL4	WD40‐TPR/TPR‐RBS
RPL10	WD40‐TPR/TPR‐RBS
RPL14	WD40‐TPR/TPR‐RBS
RPL17	WD40‐TPR/TPR‐RBS
RPL18A	WD40‐TPR/TPR‐RBS
RPL22	WD40‐TPR/TPR‐RBS
RPL24	WD40‐TPR/TPR‐RBS
RPL27A	WD40‐TPR/TPR‐RBS
RPL30	WD40‐TPR/TPR‐RBS
RPSA	WD40‐TPR/TPR‐RBS
RPS2	WD40‐TPR/TPR‐RBS
RPS4X	WD40‐TPR/TPR‐RBS
RPS6	WD40‐TPR/TPR‐RBS
RPS7	WD40‐TPR/TPR‐RBS
RPS8	WD40‐TPR/TPR‐RBS
RPS9	WD40‐TPR/TPR‐RBS
RPS11	WD40‐TPR/TPR‐RBS
RPS13	WD40‐TPR/TPR‐RBS
RPS18	WD40‐TPR/TPR‐RBS
RPS23	WD40‐TPR/TPR‐RBS
RPS25	WD40‐TPR/TPR‐RBS
RPS26	WD40‐TPR/TPR‐RBS
RPS27	WD40‐TPR/TPR‐RBS
RPL8	WD40‐TPR
RPL9	WD40‐TPR
RPL13	WD40‐TPR
RPL18	WD40‐TPR
RPL28	WD40‐TPR
RPL38	WD40‐TPR
RPLP2	WD40‐TPR
RPS5	WD40‐TPR
RPS10	WD40‐TPR
RPS24	WD40‐TPR
RPL3	TPR‐RBS
RPL5	TPR‐RBS
RPL23A	TPR‐RBS
RPL26	TPR‐RBS
RPL27	TPR‐RBS
RPL31	TPR‐RBS
RPLP0P6	TPR‐RBS
RPS15A	TPR‐RBS

^a^ The oligomerization‐dependent interaction of these proteins with the juxtaposed WD40 repeats–TPR domains, or the TPR‐RBS domains is described in Francisco‐Velilla, Abellan, Embarc‐Buh et al. 2024.

**TABLE 2:** Proteins involved in splicing, RNA stability, and translation regulation associated with GEMIN5 oligomerization domains.

Protein name	Biological processes	GEMIN5 interacting domains^a^
DHX15	Splicing	WD40‐TPR/TPR‐RBS
EFTUD2	Splicing	WD40‐TPR/TPR‐RBS
FUS	Splicing	WD40‐TPR/TPR‐RBS
HNRNPF	Splicing	WD40‐TPR/TPR‐RBS
HNRNPM	Splicing	WD40‐TPR/TPR‐RBS
PRPF4	Splicing	WD40‐TPR/TPR‐RBS
PRPF6	Splicing	WD40‐TPR/TPR‐RBS
RBM14	Splicing	WD40‐TPR/TPR‐RBS
RBM17	Splicing	WD40‐TPR/TPR‐RBS
RBMX	Splicing	WD40‐TPR/TPR‐RBS
SF3B1	Splicing	WD40‐TPR/TPR‐RBS
SF3B2	Splicing	WD40‐TPR/TPR‐RBS
SF3B3	Splicing	WD40‐TPR/TPR‐RBS
SNRNP70	Splicing	WD40‐TPR/TPR‐RBS
SNRNPB2	Splicing	WD40‐TPR/TPR‐RBS
SRSF1	Splicing	WD40‐TPR/TPR‐RBS
SRSF3	Splicing	WD40‐TPR/TPR‐RBS
TRA2A	Splicing	WD40‐TPR/TPR‐RBS
CDC5L	Splicing	WD40‐TPR
DDX23	Splicing	WD40‐TPR
DDX41	Splicing	WD40‐TPR
ESS2	Splicing	WD40‐TPR
HNRNPA2B1	Splicing	WD40‐TPR
HNRNPA3	Splicing	WD40‐TPR
KHSRP	Splicing	WD40‐TPR
NONO	Splicing	WD40‐TPR
PRPF19	Splicing	WD40‐TPR
PRPF3	Splicing	WD40‐TPR
PRPF31	Splicing	WD40‐TPR
RBM10	Splicing	WD40‐TPR
RBM39	Splicing	WD40‐TPR
SART1	Splicing	WD40‐TPR
THRAP3	Splicing	WD40‐TPR
U2AF2	Splicing	WD40‐TPR
ZCCHC8	Splicing	WD40‐TPR
AKAP8L	Splicing	TPR‐RBS
FUBP2	Splicing	TPR‐RBS
HNRNPU	Splicing	TPR‐RBS
RAVER1	Splicing	TPR‐RBS
SFPQ	Splicing	TPR‐RBS
SNRPA	Splicing	TPR‐RBS
SNRPA1	Splicing	TPR‐RBS
SNRPD2	Splicing	TPR‐RBS
SNRPG	Splicing	TPR‐RBS
SNRPGP15	Splicing	TPR‐RBS
SNSPE	Splicing	TPR‐RBS
SRSF4	Splicing	TPR‐RBS
SRSF6	Splicing	TPR‐RBS
SRSF9	Splicing	TPR‐RBS
TAF15	Splicing	TPR‐RBS
TRA2B	Splicing	TPR‐RBS
GEMIN2	Splicing/SMN complex	WD40‐TPR/TPR‐RBS
GEMIN3	Splicing/SMN complex	WD40‐TPR/TPR‐RBS
GEMIN4	Splicing/SMN complex	WD40‐TPR/TPR‐RBS
GEMIN5	Splicing/SMN complex	WD40‐TPR/TPR‐RBS
GEMIN6	Splicing/SMN complex	WD40‐TPR/TPR‐RBS
GEMIN8	Splicing/SMN complex	WD40‐TPR/TPR‐RBS
SMN	Splicing/SMN complex	WD40‐TPR/TPR‐RBS
UNRIP	Splicing/SMN complex	WD40‐TPR/TPR‐RBS
DDX1	Splicing/RNA stability	WD40‐TPR/TPR‐RBS
HNRNPC	Splicing/RNA stability	WD40‐TPR/TPR‐RBS
TARDBP	Splicing/RNA stability	WD40‐TPR/TPR‐RBS
SYNCRIP	Splicing/RNA stability/Translation regulation	WD40‐TPR/TPR‐RBS
YBX1	Splicing/RNA stability/Translation regulation	WD40‐TPR/TPR‐RBS
FMR1	Splicing/Translation regulation	TPR‐RBS
FXR1	Splicing/Translation regulation	TPR‐RBS
FXR2	Splicing/Translation regulation	TPR‐RBS
MOV10	RNA stability	WD40‐TPR/TPR‐RBS
UPF1	RNA stability	WD40‐TPR/TPR‐RBS
GIGYF2	RNA stability/Translation regulation	WD40‐TPR/TPR‐RBS
HNRNPD	RNA stability/Translation regulation	WD40‐TPR/TPR‐RBS
IG2BP2	RNA stability/Translation regulation	WD40‐TPR/TPR‐RBS
PABPC4	RNA stability/Translation regulation	WD40‐TPR/TPR‐RBS
YBX3	RNA stability/Translation regulation	WD40‐TPR/TPR‐RBS
YTHDF1	RNA stability/Translation regulation	WD40‐TPR/TPR‐RBS
YTHDF2	RNA stability/Translation regulation	WD40‐TPR/TPR‐RBS
YTHDF3	RNA stability/Translation regulation	WD40‐TPR/TPR‐RBS
ATXN2	Translation regulation	WD40‐TPR/TPR‐RBS
ATXN2L	Translation regulation	WD40‐TPR/TPR‐RBS
CAPRIN1	Translation regulation	WD40‐TPR/TPR‐RBS
DDX3X	Translation regulation	WD40‐TPR/TPR‐RBS
EIF3D	Translation regulation	WD40‐TPR/TPR‐RBS
FUBP3	Translation regulation	WD40‐TPR/TPR‐RBS
G3BP1	Translation regulation	WD40‐TPR/TPR‐RBS
ILF3	Translation regulation	WD40‐TPR/TPR‐RBS
PRRC2C	Translation regulation	WD40‐TPR/TPR‐RBS
UBAP2L	Translation regulation	WD40‐TPR/TPR‐RBS
AGO2	Translation regulation	TPR‐RBS
EIF4E	Translation regulation	TPR‐RBS
ELAVL1	Translation regulation	TPR‐RBS
EPRS	Translation regulation	TPR‐RBS
LARP1	Translation regulation	TPR‐RBS
NUDT21	Translation regulation	TPR‐RBS
PURA	Translation regulation	TPR‐RBS
UBA52	Translation regulation	TPR‐RBS
BYSL	rRNA processing	WD40‐TPR
DDX21	rRNA processing	WD40‐TPR
EXOSC10	rRNA processing	WD40‐TPR
EXOSC5	rRNA processing	WD40‐TPR
EXOSC7	rRNA processing	WD40‐TPR
EXOSC9	rRNA processing	WD40‐TPR
MTREX	rRNA processing	WD40‐TPR
NVL	rRNA processing	WD40‐TPR

^a^ The oligomerization‐dependent interaction of these proteins with the juxtaposed WD40 repeats –TPR domains, or the TPR–RBS domains is described in Francisco‐Velilla, Abellan, Embarc‐Buh et al. 2024.

Collectively, the large modifications of the protein interactome observed as a function of the modular arrangement used to capture the factors associated with GEMIN5 suggest the assembly of distinct macromolecular architectures involved in proteome association, and therefore in protein function (Francisco‐Velilla, Abellan, Embarc‐Buh et al. 2024). Major changes are observed in the networks preferentially interacting with the oligomerization‐deficient G5N repeats region, which include Splicing and Protein folding (Figure 2). The latter comprises almost all members of the T‐complex protein Ring Complex (TRIC complex). On the other hand, analysis of the interactome associated with GEMIN5 mutants found in patients leading to dimerization problems (Francisco‐Velilla, Embarc‐Buh, del Caño‐Ochoa et al. 2022), as well as those affecting the phosphorylation state of residues within the TPR moiety (Francisco‐Velilla, Embarc‐Buh, Abellan et al. 2022) revealed strong interference with the networks preferentially bound to GEMIN5.

Analogous to GEMIN5, numerous RBPs contain oligomerization motifs that regulate their state and, consequently, their function (Sanchez et al. 2014; Lou et al. 2022). In particular, the dimerization domain of the SMN protein modulates the assembly of the entire complex depending upon the phosphorylation state of its tyrosine glycine (YG) motif (Gupta et al. 2015; Gupta et al. 2021). The assembly of macromolecular complexes is usually assisted by chaperones, a feature that can also be attributed to the SMN protein (Matera et al. 2024). This recent article provides evidence for the chaperoning activity of *Drosophila* SMN protein in fly lines, showing a prominent connection of SMN with heat‐shock chaperones (HSPs), and also with histone/nucleosome assembly chaperones. Curiously, the SMN interactome partially overlaps with that of GEMIN5 (Francisco‐Velilla, Abellan, Embarc‐Buh et al. 2024). The possibility that this could be due to recently reported GEMIN5‐SMN protein–protein interaction (Fortuna et al. 2023) remains to be analyzed in future studies.

## 4 Ribosome Binding

In line with the involvement of GEMIN5 in the regulation of translation, the protein is detected in native ribosomal fractions prepared from human cells (Francisco‐Velilla et al. 2016), and also forms part of the list of ribosomal associated proteins (RAPs) found by alternative approaches (Simsek et al. 2017). GEMIN5 is readily detected in native ribosomes, but not in salt‐washed ribosomes, demonstrating that it is not an integral part of the ribosome. Furthermore, the interaction of GEMIN5 with ribosomes is abrogated by specific WD40 repeats mutants, suggesting that the overall architecture of the G5N region is important for ribosome binding. Detailed analysis of specific ribosome components indicates that the 60S ribosomal proteins RPL4 and RPL3 mediate the interaction of GEMIN5 with the ribosome. These proteins coprecipitate with GEMIN5 in GST‐pull downs (Francisco‐Velilla et al. 2016), suggesting that GEMIN5 can form a binary complex with these proteins independent of RNA bridges. Interestingly, RPL3 and RPL4 are conserved proteins with terminal extensions located on the solvent side of the 80S ribosome (Yusupova and Yusupov 2014). In particular, RPL3 coordinates the binding of elongation factors and aminoacylated tRNAs, whereas RPL4 is located within the peptide tunnel (Meskauskas and Dinman 2007; Mailliot et al. 2016). Taken together, these data lead to the belief that the interaction of GEMIN5 with RPL3 and RPL4 may interfere with translation elongation (Francisco‐Velilla et al. 2016).

Regarding the function of GEMIN5 in global protein synthesis, the polysome profiles are affected by GEMIN5 depletion or overexpression, concomitant with an increase or a decrease, respectively, in global protein synthesis. Under normal conditions, the protein is immunodetected in the light fractions of a polysome profile entering up to the 80S peak, as also occurs for the translation initiation factor eIF3b. Although with lower intensity, GEMIN5 is detected in the heavy polysome fractions (Francisco‐Velilla et al. 2016). However, overexpression of GEMIN5 shifts the signal to the heavy polysomes, suggesting that the protein may have a stalling effect on translation elongation, in agreement with its capacity to modulate negatively global protein synthesis (Pacheco et al. 2009). A more recent study discovered that the translation down‐regulatory function is linked to the presence of both the WD40 repeats domain and the TPR module, since only proteins carrying both of these domains are clearly detected in heavy polysomes (Francisco‐Velilla, Abellan, Embarc‐Buh et al. 2024). Therefore, it is inferred that polysome association requires protein oligomerization but also the G5N architecture (Xu et al. 2016), in accordance with the observation that the N‐terminal region of the protein is sufficient to bind the 60S ribosomal subunit (Francisco‐Velilla et al. 2016) and a large number of ribosomal proteins (Table 1).

The notion of heterogeneous ribosome populations with regard to protein synthesis regulation is connected to distinct types of evidence, including the dynamics of ribosomal proteins but also the presence of RAPs in the ribosome population. The existence of ribosome subpopulations is exemplified by the effect of the dynamic ribosome composition of specific proteins (RACK1, or RPLP1 and RPLP2) on protein synthesis (Remacha et al. 1995; Martínez‐Azorín et al. 2008; Majzoub et al. 2014; Campos et al. 2017). Furthermore, the notion of distinct ribosome populations aligns well with the involvement of ribosome heterogeneity in development and disease (Chouaib et al. 2020; Bourke et al. 2023; Gao and Wang 2024). Remarkably, not only is GEMIN5 associated with ribosomes, but the protein SMN is also associated with ribosomes and proteins directly involved in translation regulation (Lauria et al. 2020), expanding the list of SMN members connected to translation regulatory events (Sanchez et al. 2013; Bernabò et al. 2017). Nevertheless, understanding the mechanistic basis of the direct role of RAPs in the regulation of protein synthesis awaits further investigations.

The discovery of RAPs in different tissues underscores new functions of a heterogeneous population of ribosomes (Chen et al. 2014; Shi et al. 2017; Li and Wang 2020; Susanto et al. 2024), possibly regulating translation elongation during neural development and immune activation. Similar to GEMIN5, there are examples of proteins carrying TPR‐like motifs, such as ELAV‐like RNA‐binding protein 2 (ELAVL2) and several Interferon (IFN)‐induced proteins, that bind both the mRNA and the ribosome. However, although TPR motifs are present in many RBPs, the interplay between these specific domains and the ribosome remains unknown.

## 5 Regulation of Protein Synthesis

Research done over the years identified a large number of GEMIN5 partners, both RNAs and proteins, compatible with its involvement in translation regulatory events (Pacheco et al. 2009; Workman et al. 2015; Francisco‐Velilla et al. 2016; Van Nostrand, Pratt et al. 2020), and also expanded the previously identified function in snRNP assembly (Battle et al. 2006; Yong et al. 2010).

### 5.1 mRNA Targets

**FIGURE 3:**
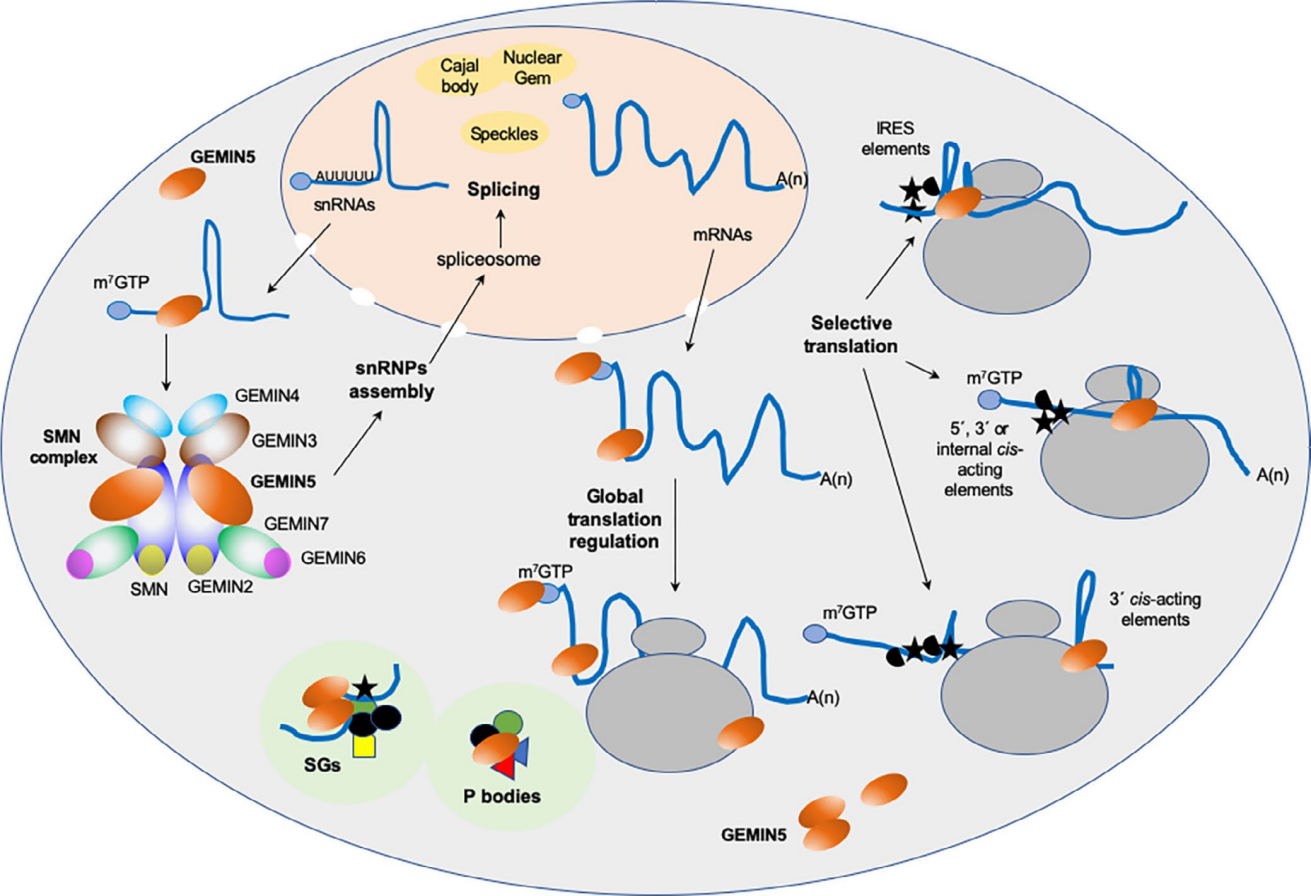
Overview of GEMIN5 partners and its involvement in snRNPs assembly and translation regulatory events. Molecules (RNAs, proteins, and ribosomes) interacting with GEMIN5 (dark orange circle) in the cytoplasm are schematically depicted. Recognition of the Sm site (5′AUUUUU) of snRNAs in the cytoplasm by GEMIN5 allows the delivery and assembly of snRNP particles by the SMN macromolecular complex (SMN and GEMIN2‐8) (Battle et al. 2006; Fischer et al. 2011). snRNPs are then transported back to the nucleus, where they participate in RNA splicing (Wahl et al. 2009). Within the nuclear compartment, the SMN complex is located in Cajal bodies, nuclear gems and speckles (Gubitz et al. 2002), although the detection of GEMIN5 in distinct nuclear granules has been controversial (Battle et al. 2007; Le Hao et al. 2021). The m^7^GTP cap structure (solid light blue circle) found at the 5′ end of snRNAs and mRNAs is also recognized by the WD40 repeats domain located in the N‐terminus of GEMIN5 (Jin et al. 2016; Xu et al. 2016). In addition, independently of the SMN complex, GEMIN5 binds with native ribosomes (Francisco‐Velilla et al. 2016), regulating cap‐dependent translation. The association of GEMIN5 with the 60S ribosomal subunit through interaction with RPL3 and RPL4 is schematically represented. Regarding the interaction with mRNAs, GEMIN5 binds directly with viral IRES elements, regulating cap‐independent initiation of protein synthesis in concerted action with eIFs and other RBPs (depicted by black stars and semicircles) (Pacheco et al. 2009). Cap‐dependent selective translation of mRNAs carrying 5′, internal, or 3′ *cis*‐acting elements, illustrated by the 5′ terminal oligopyrimidine (TOP), the stem‐loop 1 of *GEMIN5* mRNA, or the 3′ UTR of *SMN* mRNA, has been reported (Embarc‐Buh et al. 2022; Francisco‐Velilla et al. 2018; Workman et al. 2015). Preferential translation of non‐polyadenylated histone mRNAs via 3′ *cis*‐acting elements (histone stem‐loop, HSL) bound to GEMIN5, or as a ribosome‐associated protein was previously shown (Embarc‐Buh et al. 2022). Various proteins (colored circles, triangles, and squares) interact with GEMIN5 as a function of the oligomerization state (Francisco‐Velilla, Abellan, Embarc‐Buh et al. 2024), either in the soluble cytoplasmic fraction, or in protein condensates forming membrane‐less granules, stress granules (SGs) and P bodies (Berchtold et al. 2018; Vu et al. 2021; Delle Vedove et al. 2022).

The diverse activities of GEMIN5 reviewed here rely on the capacity to recognize multiple partners through different functional domains. Beyond the ribosomal proteins mentioned above (Table 1), well‐documented partners include several proteins involved in translation regulation (Table 2) and various types of RNA structural motifs (Figure 3), exemplified by the snRNAs recognized by the WD40 repeat motifs at the N‐terminal end (Battle et al. 2006) and the internal ribosome entry site (IRES) element recognized by the RNA‐binding site RBS1 at the C‐terminal end (Fernandez‐Chamorro et al. 2014).

Concerning the variety of RNA targets, the GEMIN5 protein has been identified in association with several viral and cellular mRNAs under different physiological situations (Pacheco et al. 2009; Piñeiro et al. 2013; Francisco‐Velilla et al. 2018; Garcia‐Moreno et al. 2019; Van Nostrand, Pratt et al. 2020; Kamel et al. 2021). During the search for RBPs upregulated in Sindbis virus (SINV) infected cells by RNA‐interactome capture, GEMIN5 was identified within the subset of proteins bound to the 5′ end of viral RNAs, regulating viral gene expression. Upregulated expression of GEMIN5, but not any other SMN member, is strongly stimulated by SINV infection. The protein is redistributed to the viral factories, co‐localizing with SINV RNA and various viral proteins, indicating a specific GEMIN5 response to viral infection. Indeed, overexpression of GEMIN5 in SINV‐infected cells inhibits viral capsid synthesis (Garcia‐Moreno et al. 2019), presumably preventing translation by interfering with ribosomal function. Accordingly, protein–protein interaction analysis performed in SINV‐infected cells indicates that GEMIN5 is associated with the 60S ribosomal subunit, in full agreement with previous data showing that GEMIN5 impacts global protein synthesis at the translation elongation step through its direct interaction with the 60S ribosomal subunit (Francisco‐Velilla et al. 2016). Crosslinking immunoprecipitation followed by sequencing (iCLIP) shows that the highest coverage map within the 5′ end of the genomic RNA (gRNA) and subgenomic RNA (sgRNA), and also with the downstream stem‐loop that stimulates the translation of the sgRNA (Frolov and Schlesinger 1996). These data support a model in which GEMIN5 recognizes the 5′ end of both the genomic and the subgenomic viral RNA, regulating SINV viral protein synthesis.

The complexity of GEMIN5 functions in gene expression pathways was at least in part uncovered from global approaches aimed to identify its targets in the cellular context. Overall, the results derived from CLIP‐based methodologies suggest that GEMIN5 acts as a platform, serving as a hub for distinct RNA‐protein networks (Van Nostrand, Pratt et al. 2020). Specifically, the mRNA hits of the full‐length GEMIN5 are enriched in the 5′ UTR around the cap and the initiation codon. In contrast to the full‐length protein, a prominent feature of the cellular hits of the RBS1 domain consists of G:C‐rich sequences predicted to fold as a stem longer than 10 bp (Francisco‐Velilla et al. 2018). The RBS1 domain consists of an intrinsically disordered region (IDR) which, however, contains conserved positively charged and aromatic residues necessary for RNA binding, followed by a helical region (Embarc‐Buh et al. 2021). Among the RNA targets of the RBS1 region, the most abundant hit is a predicted thermodynamically stable stem‐loop of *GEMIN5* mRNA. Biochemical and functional characterization of this RNA target demonstrates that RBS1 physically interacts with its own mRNA in the cellular cytoplasm (Francisco‐Velilla et al. 2018), and in vitro using purified RBS1 protein as well (Francisco‐Velilla et al. 2020). These data also indicate that the flanking sequences of the hairpin enhance RBS1 binding, presumably by stabilizing the optimal structure required for protein recognition. Importantly, RBS1 stimulates the translation of *GEMIN5* mRNA (Francisco‐Velilla et al. 2018), providing a regulatory feedback loop that counteracts the negative effect of GEMIN5 on global translation. This regulatory mechanism gives support to the hypothesis that the coevolved RNA‐protein interaction (Francisco‐Velilla et al. 2020) presumably fine‐tunes the availability of GEMIN5 to play its various roles in gene expression control.

### 5.2 Translation Initiation

In eukaryotic cells, translation initiation in the vast majority of mRNAs occurs by a cap‐dependent mechanism, through the recognition of the m^7^GTP residue placed at the 5′ end of the mRNA by the cap‐binding protein eIF4E (Hinnebusch et al. 2016). Actually, translation initiation is regulated by *cis*‐acting RNA elements and *trans*‐acting factors, including several translation initiation factors (eIFs) and multiple RBPs. Beyond the large majority of mRNAs using a cap‐dependent mechanism, specific subsets of mRNAs that overcome cap‐dependent inhibition use 5′ end‐independent mechanisms (Sonenberg and Hinnebusch 2009; Akirtava and McManus 2021). A well‐documented example of a 5′ end‐independent mechanism is driven by the IRES elements, initially discovered in picornavirus RNAs (Pelletier and Sonenberg 1988; Jang et al. 1988). Most IRES elements are heavily structured RNA entities (Fernandez et al. 2011; Plank and Kieft 2012; Mailliot and Martin 2018) that can function independently in heterologous contexts, recruiting eIFs and ribosomal subunits to an internal position in the mRNA (Martinez‐Salas et al. 2018). This cap‐independent mechanism depends on RNA structural elements and *trans*‐acting factors (Lozano and Martínez‐Salas 2015; Lee et al. 2017; Barrera et al. 2020).

Research carried out in cells infected with positive‐strand RNA viruses underscores the inactivation of the normal function of RBPs by proteolysis, modification of the posttranslational state, and/or redistribution of RBPs in the cellular compartment (Saiz and Martinez‐Salas 2021; Iselin et al. 2022). A classic example is illustrated by the cleavage of the translation initiation factor eIF4G by picornavirus proteases (reviewed by Sonenberg and Hinnebusch 2009). Cleavage of eIF4G results in the separation of critical regions of the protein, eventually abrogating cap‐dependent translation (Gradi et al. 2004). Following cleavage by viral proteases, the poly(A) binding protein (PABP) and the eIF4E (the cap‐binding factor) binding domains remain at the N‐terminal cleavage product of the eIF4G protein, while the C‐terminal product, harboring eIF4A binding sites, is sufficient to direct the initiation of viral protein synthesis via the IRES element (López de Quinto and Martínez‐Salas 2000).

GEMIN5 was serendipitously identified in the course of RNA‐protein investigations using UV‐crosslink assays followed by mass spectrometry analysis as one of the RBPs bound to specific structural domains of viral IRES elements (Pacheco et al. 2009) (Figure 3). Subsequent work discovered that GEMIN5 was proteolyzed by the leader protease (L^pro^) of the picornavirus foot‐and‐mouth disease virus (FMDV). This proteolytic cleavage generates a C‐terminal product of 85 kDa that encompasses the entire G5C region, enhancing IRES activity (Piñeiro et al. 2012). Importantly, the upregulation of IRES‐driven protein synthesis by G5C is opposed to the effect exerted by the full‐length protein, which negatively regulates IRES‐dependent translation (Pacheco et al. 2009).

It is worth mentioning that the unexpected discovery of GEMIN5 proteolytic cleavage in FMDV‐infected cells leading to the detection of the G5C fragment opened new avenues to investigate its functional relevance in the context of normal cell physiology, as evidenced by studies performed with different versions of the protein carrying the TPR module and the RBS domain (Piñeiro et al. 2013; Fernandez‐Chamorro et al. 2014). The translation‐enhancing behavior of G5C compared to G5N (Francisco‐Velilla et al. 2018; Moreno‐Morcillo et al. 2020) is presumably due to differences in the macromolecular interactome associated with each of these versions of the protein. In accordance with this hypothetical idea, the differential effect in translation regulation of the oligomerization‐proficient fragment G5C could be due to the loss of translation repression induced by reduced levels of the free endogenous GEMIN5, likely caused by G5C‐GEMIN5 heterodimer formation (Figure 1C).

In conclusion, GEMIN5 can regulate translation in different manners by using distinct functional domains. The negative regulatory role of the global translation of the N‐terminal region is likely related to the ribosome binding capability of G5N and to the sedimentation with heavy polysomes (Francisco‐Velilla et al. 2016). Accordingly, the WD40 repeats domain located in this region recognizes the cap structure (Xu et al. 2016), consistent with data showing GEMIN5 capture in cap‐sepharose beads (Bradrick and Gromeier 2009). In contrast, G5C enhances IRES‐dependent translation through the direct interaction with a stable viral RNA hairpin flanked by A/U/C‐rich sequences (Piñeiro et al. 2013).

### 5.3 Selective Translation

Selective translation allows orchestration of the synthesis of specific proteins through the action of *cis*‐acting elements of mRNAs and individual RBPs (Zhulyn et al. 2023; Tidu and Martin 2024). In agreement with this idea, the presence of certain RNA motifs in a given mRNA provides a signature for the interaction with specific proteins (Gerstberger et al. 2014; Wassmer et al. 2024). In turn, the interplay between *cis*‐acting elements on the mRNA and regulatory proteins allows selective translation of specific mRNA subsets, sustaining the maintenance of cell integrity in all living organisms by rapid modification of its proteome. Furthermore, this regulatory mechanism allows cells to adapt their metabolism to the physiological environment.

Several types of evidence reinforce the idea that GEMIN5 regulates translation of selective mRNAs through the recognition of specific RNA motifs, located in different regions of the mRNA (Workman et al. 2015; Francisco‐Velilla et al. 2018; Garcia‐Moreno et al. 2019) (Figure 3). In the particular case of SMN mRNA, GEMIN5 binds in the 3′ UTR immediately upstream of the poly(A) tail through a sequence that is reminiscent of the snRNP code of snRNAs (Workman et al. 2015). The snRNP code consists of a bipartite site containing the Sm site (5′ AUUUUU) near a stem‐loop either 5′ or 3′ of the Sm site (Golembe et al. 2005). Given that previous work reported the selective translation of 80S monosomes versus polysomes (Heyer and Moore 2016), the shift of SMN mRNA from heavy polysomes to light polysomes and monosomes induced in cells with decreased levels of GEMIN5 suggests that this protein functions as an activator of SMN translation. On the other hand, analysis of the GEMIN5 complexes retrieved with the SMN mRNA indicates the absence of the SMN protein, strongly suggesting that the regulatory activity is solely due to GEMIN5. This observation fits well with previous data showing that a large fraction of GEMIN5 exists in the cytoplasm in SMN‐free complexes (Battle et al. 2007). Nevertheless, since SMN also has a role in trafficking and mRNA transport (Donlin‐Asp et al. 2016; Chaytow et al. 2018), the possibility that SMN and GEMIN5 proteins may cooperate jointly cannot be discarded, likely regulating axonal gene expression in animal models.

The differential function of GEMIN5 in selective translation is further illustrated by the mRNAs associated with polysomes in a GEMIN5‐dependent manner (Embarc‐Buh et al. 2022). Remarkably, the mRNAs that appear to be underrepresented in polysomes in *GEMIN5‐silenced* cells encode proteins that participate in translation regulation and nucleic acid metabolism. In contrast, the overrepresented processes encoded by the GEMIN5‐repressed mRNAs are related to signaling processes. Generally, the mRNAs primarily enriched in polysomes in a GEMIN5‐dependent manner carry unique *cis*‐acting elements, the 5′ terminal oligopyrimidine tracts (TOP) (Meyuhas and Kahan 2015; Fonseca et al. 2018), and the histone stem‐loop (hSL) structure at the 3′ end of the mRNA (Marzluff and Koreski 2017; Dominski and Tong 2021) (Figure 3). These mRNAs encode ribosomal proteins and histones, respectively, which are essential factors absolutely required for protein synthesis and nuclear metabolism. Therefore, the ability of GEMIN5 to promote the translation of these mRNAs suggests a specific regulatory role in fundamental cellular processes that need to be coordinated to preserve cell growth. These results are fully consistent with the involvement of GEMIN5 in the proliferation of zebrafish hematopoietic stem progenitor cells (Liu et al. 2021).

Concerning the role of GEMIN5 in selective translation of histone mRNAs, it has been recently reported that flunarizine treatment (a calcium channel blocker used to treat neurological diseases) (Younis et al. 2010) produces a transient increase in GEMIN5 protein levels followed by a gradual increase in mRNA levels encoding the *lysine demethylase 6b* (*Kdm6b*), independently of SMN protein levels (Salman et al. 2024). In mice, KDM6B is expressed in the spinal cord during neonatal development (Liau et al. 2023), and a recent report indicates that the loss of function of the *Kdm6b* gene is associated with neurodevelopmental disorders (Rots et al. 2023). Since GEMIN5 positively regulates translation of histone mRNAs (Embarc‐Buh et al. 2022), it can be hypothesized that dysregulated epigenetic marks might contribute to the pathological phenotype of *GEMIN5* variants as well. Furthermore, it is also possible that mutations in the *GEMIN5* gene could result in altered levels of both GEMIN5 and KDM6B, perturbing the normal deposition of histone marks and, consequently, the progression of gene expression programs during embryonic and postnatal development.

## 6 Additional Functions

### 6.1 Alternative Splicing

As mentioned before, GEMIN5 is responsible for the recognition of the Sm code in snRNAs and the assembly of snRNPs (Lau et al. 2009; Yong et al. 2010), the principal components of the spliceosome (Kastner et al. 2019). Thus, it was plausible to hypothesize that altered levels of GEMIN5 could affect RNA splicing events. The use of different splice sites in any given pre‐mRNA gives rise to mRNAs by alternative splicing (AS), a process that is known to generate a large degree of RNA variability (Rogalska et al. 2023; Carrocci and Neugebauer 2024). This process also contributes to proteome diversity due to differences in the coding region of the resulting alternatively spliced mRNAs (Smith and Valcárcel 2000). Previous data suggested that substitutions within G5N could perturb snRNA binding (Battle et al. 2006; Xu et al. 2016; Jin et al. 2016), presumably disrupting the function of the SMN complex in snRNP assembly and thereby spliceosome activity (Fischer et al. 2011; Coady and Lorson 2011). In agreement with this possibility, recent evidence indicates that clinical *GEMIN5* variants, as well as protein upregulation, jeopardize alternative splicing (Fortuna et al. 2023; Francisco‐Velilla, Abellan, Garcia‐Martin et al. 2024). Along with this idea, amino acid changes in other regions of GEMIN5 strongly reduce protein stability (Kour et al. 2021; Francisco‐Velilla, Embarc‐Buh, del Caño‐Ochoa et al. 2022), likely resulting in a global decrease in spliceosomal activity.

**FIGURE 4:**
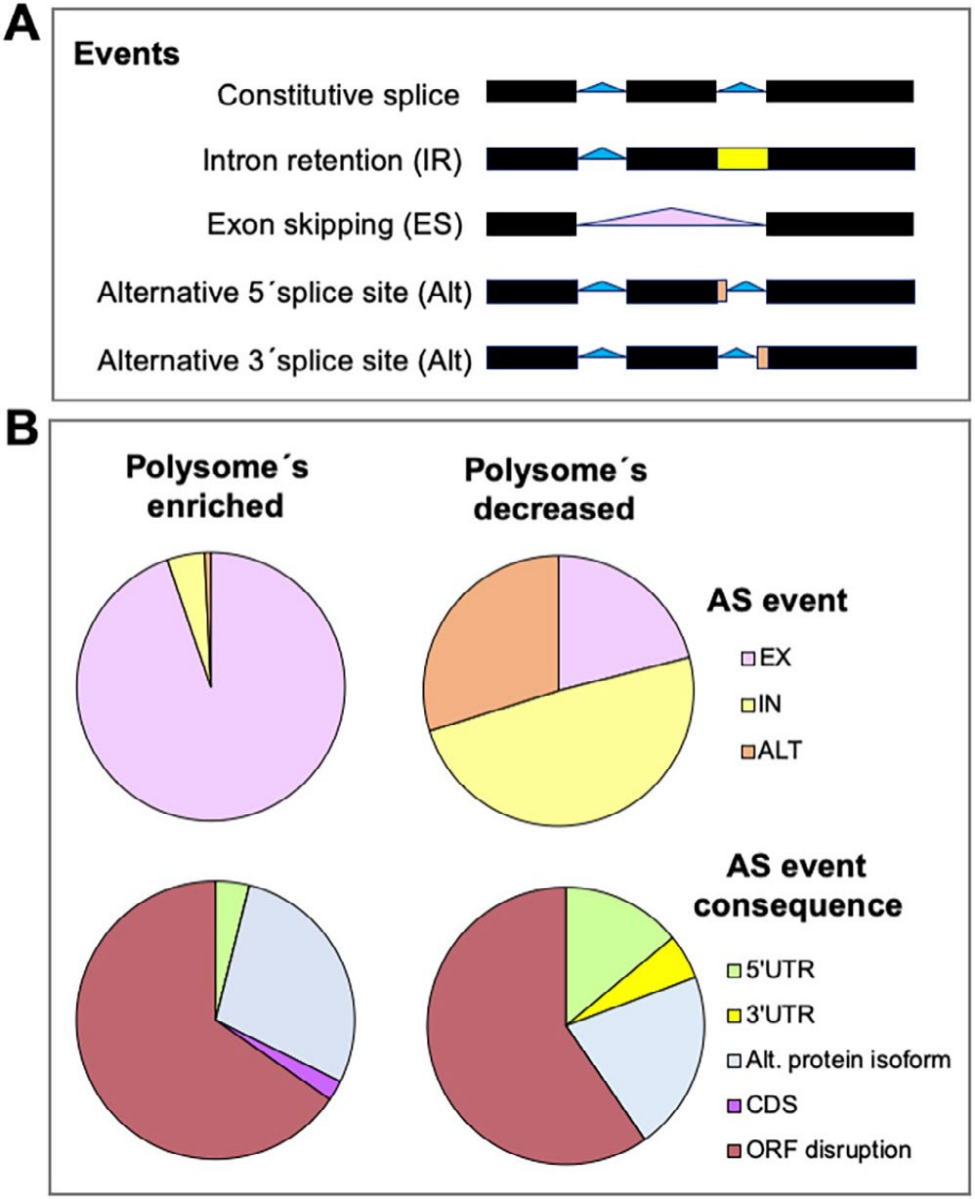
Overview of the impact of altered levels of GEMIN5 on alternative splicing. (A) Schematic cartoon representing splicing events. The most frequent constitutive splice events in pre‐mRNAs are depicted by solid blue triangles. Main alternative splicing (AS) events are indicated: (intron retention (IR, yellow), exon skipping (ES, pink), and alternative 5′ or 3′ splice site (Alt, orange). (B) Summary of mRNAs carrying distinct AS events differentially engaged into polysomes. Distribution of mRNAs carrying AS events enriched in polysomes (left panel), or decreased in polysomes (right panel), identified in HEK293 cells with high levels of GEMIN5 protein relative to control cells. The pie charts show the type of event (ES, IR, or Alt) or the consequences predicted on the proteome (ORF disruption, alternative protein isoform, coding sequence, the 5′ UTR or the 3′ UTR). The mRNAs were identified in the heavy polysome fractions of sucrose gradients followed by RNA‐Seq and Vast‐tools splicing analyses (Francisco‐Velilla, Abellan, Garcia‐Martin et al. 2024).

Among the splicing events naturally occurring in human cells (Figure 4A), the AS profile of a defective *GEMIN5* variant shows that exon skipping represents the largest percentage of AS events (Fortuna et al. 2023), mainly affecting the processing of pre‐mRNAs involved in biological pathways related to DNA metabolism, signaling, and transcriptional regulation. Interestingly, homozygous *GEMIN5* variants generated in induced pluripotent stem cells (iPSC)‐derived neurons exhibit a significant reduction in snRNP assembly compared to IPSC controls, resulting in global splicing defects (Kour et al. 2021). These deficiencies could be attributed to flawed mature importation of U snRNPs into Cajal bodies (CBs), since overexpression of SMN rescued snRNP‐core assembly formation in mutant *GEMIN5* iPSC neurons, in turn resulting in a significant reduction of AS events (Fortuna et al. 2023).

Beyond the repercussions of *GEMIN5* pathological mutants in splicing, altered levels of wild‐type GEMIN5 expression also result in an excess of AS events relative to normal levels of expression, with exon skipping (ES) being the most frequent type of differential AS event observed (Francisco‐Velilla, Abellan, Garcia‐Martin et al. 2024). In addition, the analysis of the consequences of AS events on the proteome predicted that the largest percentage corresponds to open reading frame (ORF) disruption, followed by alternative protein isoforms, while reorganization of the 5′ and 3′ UTRs represented a very small percentage. Remarkably, RNA‐Seq identification of the mRNAs associated with heavy polysomes under conditions of increased levels of GEMIN5 indicated that a large fraction of the AS mRNAs was engaged in translationally active ribosomes (Francisco‐Velilla, Abellan, Garcia‐Martin et al. 2024) (Figure 4B). Besides, the AS events in genes leading to ORF disruption under conditions of GEMIN5 upregulation encode proteins impacting key cellular processes, affecting distinct steps of cell proliferation, including splicing, ribosome biogenesis, or RNA stability. This observation is fully consistent with the relevance of maintaining physiological levels of this protein.

The broad range of mRNAs comprising AS events engaged in polysomes upon GEMIN5 upregulation supports the notion that the protein has evolved as a regulator of gene expression, consistent with its dual role as a member of the SMN complex and as a modulator of protein synthesis, ultimately impinging on cell homeostasis. Along with this view, altering the protein levels, either increased or decreased, dysregulates the normal activity of GEMIN5 in translation regulation and splicing (Embarc‐Buh et al. 2022; Francisco‐Velilla, Abellan, Garcia‐Martin et al. 2024). Although how GEMIN5 upregulation takes over the abnormal consequences on AS splicing remains unknown, it is tempting to hypothesize that overexpression of GEMIN5 can threaten the assembly of the SMN complex, favoring the assembly of intermediate complexes with GEMIN3 and GEMIN4 (Battle et al. 2007). On the other hand, it is also possible to envision that the protein–protein interaction of GEMIN5–SMN reported by Fortuna et al. (2023) could hijack part of the SMN protein in an unproductive complex.

### 6.2 Ribonucleoprotein Complexes Partnership

The diversity of partners found over the years for this protein under different physiological situations argues in favor of its contribution to the arrangement of diverse RNP complexes. Specifically, the involvement of GEMIN5 in processes related to snRNAs metabolism has been supported by different, partially controversial, observations (Shpargel and Matera 2005; Cauchi et al. 2010). The work by Hao le et al. (2007) reported that GEMIN5 is associated with SMN from cytoplasmic extracts, but not from nuclear extracts, and also that GEMIN5 is scarcely detectable in CBs. The discovery of the association of GEMIN5 with U1 and U1A snRNAs in P bodies in SMN‐deficient cells suggests the involvement of this protein in the destruction of unassembled U1 snRNAs (Jiang et al. 2018). Since P bodies are thought to be reservoirs for transcriptionally silenced mRNA (Standart and Weil 2018), it can be hypothesized that the sequestration of snRNAs from the cytoplasm into P bodies could augment the time for assembly of the heptameric Sm ring. However, more recent data indicate that defective snRNAs accumulate in P‐bodies independently of SMN and GEMIN5 proteins, but dependent on LSm1 protein (Roithová et al. 2020). These authors proposed a model by which the snRNAs that fail to acquire the Sm ring are uridylated at the 3′ end and either degraded by the 3′–5′ exoribonuclease DIS3L2, or bound by LSm1–7 proteins and sequestered together with the SMN complex to P bodies, where the Sm ring formation can be completed or the snRNAs can be degraded by the 5′–3′ exonuclease 1 (XRN1).

The interactome of any given protein analyzed under specific conditions displays the dynamic organization of cellular macromolecular complexes in response to internal and external signals (Goswami et al. 2023). Generally, the availability of RBPs and their functions is regulated in response to stress influencing the cellular proteome, such that their malfunction can result in developmental disorders (Prashad and Gopal 2021; Park et al. 2022). This is observed in neurodegenerative diseases that assemble pathological proteins into insoluble aggregates with stress granules (SG) proteins, becoming resistant to disassembly (Jia et al. 2022; Mori et al. 2024). The GEMIN5 interactome includes RBPs that form part of RNP complexes participating in multiple aspects of cell physiology (Delle Vedove et al. 2022). In full agreement with the participation of GEMIN5 in various cellular processes, the treatment of HeLa cells with sodium arsenate or heat shock resulted in the formation of SGs that contain GEMIN5, but not SMN and GEMIN2 (Battle et al. 2007). Furthermore, GEMIN5 has been identified in cytoplasmic aggregates related to stress response, viral infection, and cancer progression (Berchtold et al. 2018; Garcia‐Moreno et al. 2019; Vu et al. 2021; Wollen et al. 2021; Li et al. 2022).

In line with the data mentioned above, both the WD40 repeats domain and the TPR moiety are platforms for protein–protein interactions (Tables 1 and 2), known to be involved in a plethora of cellular processes in all organisms (Li and Roberts 2001; Xu and Min 2011). Additionally, the flexible conformation of the RBS1 domain, combined with data showing protein aggregation in the cellular context, suggests a potential role for GEMIN5 in the formation of protein condensates. The intrinsic flexibility of the RBS1 domain is essential to gain access to different RNA structural elements (Francisco‐Velilla et al. 2020). According to NMR data, the RBS1 region is preferentially unstructured with some residual secondary structure tendencies (Embarc‐Buh et al. 2021). This region of GEMIN5 binds exposed bases in RNAs using conserved aromatic and Arg residues, presumably stabilized by homodimerization processes mediated by the TPR‐like domain (Moreno‐Morcillo et al. 2020). Biomolecular condensates are frequently enriched in RBPs that contain IDRs, promoting condensate formation via liquid–liquid phase separation (LLPS) (Alberti and Dormann 2019; Wiedner and Giudice 2021). This process enables proteins to form RNA–protein condensates in membrane‐less organelles, including SGs and P‐bodies in the cytoplasm, leading to polyribosome disassembly. Although currently there are no direct evidence for LLPS in GEMIN5, the protein has been identified in cytoplasmic aggregates under rather different physiological situations (Wollen et al. 2021; Vu et al. 2021), opening the question of its contribution to SGs assembly. Curiously, siRNA perturbation of multiple genes, including *GEMIN5*, increased the fraction of cells with SGs as compared to control cells (Berchtold et al. 2018).

Collectively, the dual capacity of GEMIN5 to interact with RNAs and proteins promotes its participation in the assembly of diverse ribonucleoprotein complexes. One example is the recognition of 7S RNA by GEMIN5, presumably forming part of a complex with SMN and GEMIN2, which contributes to the biogenesis and the steady‐state level of the signal recognition particle (SRP) (Piazzon et al. 2013). SRP is involved in co‐translational targeting of secretory and membrane proteins to the endoplasmic reticulum, again providing a link between GEMIN5 and protein synthesis events. Concerning additional functions of GEMIN5 related to protein synthesis, recent data reported the identification of this protein together with other RBPs and eIFs in the list of m^7^G‐binding proteins related to the progression of several cancers (Huang et al. 2022; Li et al. 2022). In line with the potential implication of GEMIN5 in cancer progression, this protein was also identified as a mediator of m^6^A modification of mRNA, enhancing translation via eIF3 translation initiation complex recruitment in pancreatic cancer (Su et al. 2023). The potential involvement of GEMIN5 in the recognition of mRNA modifications, beyond its contribution to distinct *cis*‐acting elements, opens the repertoire on mRNA elements participating in GEMIN5‐dependent protein synthesis. Therefore, understanding the molecular mechanisms of GEMIN5 activities to maintain cell homeostasis opens new challenging areas of investigation.

## 7 Conclusion

The protein GEMIN5, initially identified as a member of the SMN complex, performs additional functions in the RNA life cycle. Understanding the biological implications of this protein in RNA and protein recognition and the molecular mechanisms of GEMIN5 action has remained obscure until the three‐dimensional structure of its individual domains was solved. The N‐terminal domain folds as two seven‐bladed ß propellers, which recognize pre‐snRNAs, while the central region adopts the conformation of a tetratricopeptide repeats module that self‐assembles into a canoe‐shaped dimer. The structural details of the dimerization domain have illuminated novel hints of this understudied protein and eventually assisted in solving the decamer architecture of the half C‐terminal region. The latter includes a non‐canonical RNA‐binding site towards the C‐terminus. Identification of the RNA targets and the protein interactors has opened new ways to explore unanticipated functions of GEMIN5, including ribosome binding and translation regulation. Furthermore, understanding the structural organization of the protein together with functional studies has brought significant implications for the role of *GEMIN5* variants in neurodevelopmental diseases. Soon after the reports defining functional and structural domains of the protein were published, it became clear that mutations in the *GEMIN5* gene detected in patients with neurological disorders could be detrimental. Patients carrying *GEMIN5* biallelic variants display neurodevelopmental delay, hypotonia, and cerebellar ataxia. Remarkably, many of these *GEMIN5* variants are predicted to generate defective proteins through changes in the TPR domain, which in turn disrupts the interaction with other cellular factors, strongly suggesting that protein dimerization and perhaps oligomerization is an essential feature of GEMIN5 to perform its diverse functions.

## Author Contributions

**Encarnacion Martinez‐Salas:** conceptualization (equal), funding acquisition (lead), supervision (equal), writing – original draft (lead). **Salvador Abellan:** investigation (equal), writing – review and editing (supporting). **Rosario Francisco‐Velilla:** investigation (equal), writing – review and editing (supporting).

## Conflicts of Interest

The authors declare no conflicts of interest.

## Related WIREs Articles

Ribosome dynamics and mRNA turnover, a complex relationship under constant cellular scrutiny.

RNA‐binding proteins in autoimmunity: from genetics to molecular biology

RNA‐binding proteins in pain

## Data Availability

Data sharing is not applicable to this article as no new data were created or analyzed in this study.
